# Research on Intelligent Target Tracking Algorithm Based on MDNet under Artificial Intelligence

**DOI:** 10.1155/2022/1550543

**Published:** 2022-04-19

**Authors:** Yu Wang

**Affiliations:** Chengyi University College, Jimei University, Information Engineering School, Xiamen 361000, China

## Abstract

Target tracking is an important subject in computer vision technology, which has developed rapidly in recent ten years, and its application have become wider and wider. In this process, it has transferred from a simple experimental tracking environment to a complex real scene where more challenges need to be solved. The rapid development of deep learning has promoted the research progress of digital vision. Target tracking technology is an important foundation of digital vision research, which makes it develop from academia to industry. In this paper, a method of target tracking using MDNet is introduced. Starting with the attention mechanism, two attention mechanisms are added to extract and integrate the better features. Case partitioning is used to reduce the investment of tracking module and minimize the network size during tracking, and its result can be prevented from getting worse. Finally, the experiment is analyzed in detail.

## 1. Introduction

Target tracking is an important subject in computer vision technology, which is widely used in human-computer interaction, vehicle navigation, automatic monitoring and so on. The general tracking problem is considered as an task of online learning, that is, to estimate the motion trajectory of subsequent targets according to the information of the first object in a video. Visualization of target tracking is a challenging task, and it is difficult to predict a large number of rapid changes well.

Generally speaking, target tracking refers to inferring the subsequent unknown target information according to the known target state information in the video sequence, and obtaining the most likely location of the target. The ability to use deep learning techniques to improve computer vision problems [1], especially in image classification [2], target detection [3], target tracking [4], semantic segmentation [5], etc.

## 2. Target Tracking Method Based on Deep Learning

Three kinds of tracking algorithms based on deep learning: depth-based tracking algorithm based on association filtering, detection-based tracking algorithm and template-based tracking algorithm [6].

Automatic tracking algorithm based on deep learning combined with correlation filtering has the same model training and prediction method as the tracking algorithm based on correlation filtering, except that the feature used is the depth feature extracted by convolutional neural network [7]. In addition, convolution features can be combined with traditional features to improve the expressive ability of features. Convolution feature is a feature of hierarchical representation, that is, deep convolution features encode more semantic features, while shallow convolution features contain fine details. By utilizing semantic class data from the profound layer and segregation data from the shallow layer, Ma prepared a straight relationship channel on a bunch of fixed convolution layers, and the coarse-to-fine technique is utilized to anticipate the area of the objective [8]. By extending the correlation filter of spatial regularization, Weber and Kanarachos et al. put forward DeepSRDCF tracker, and furthermore explored the impact of convolutional highlights on target tracking [9].

In the detection-based target tracking method, the target detection network is used to detect the target, and then, the matching calculation is utilized to relate the distinguished target frames. Ren et al. proposed a multi-target tracking method that is suitable for crowded situations. This method employ density map to predict the number of targets, detect their positions and track them. Through experiments in cells, fishes and people, it has achieved excellent tracking results. When tracking this kind of algorithm, it needs to go through two steps of detection and matching, and the matching algorithm is complicated, which makes high time complexity of this algorithm. In addition, it has strong adaptability to the changes of appearance and tracking environment [10, 11].

Target tracking technology based on template matching, which can make network evaluate the similarity of sample map and retrieval map. The most representative network architecture is Twin Network Structure [12]. Taking SiamFC as an example, the structure of twin network is introduced. The algorithm uses two branches of twin network to extract the features of sample image and image search. Then, the obtained features are applied for convolutional operation, and the response diagram is output. However, it is difficult for twin trackers such as SiamFC to distinguish similar objects, even if the objects have obvious chromatic aberration. This is because SiamFC is insensitive to the underlying characteristics [13]. By using convolutional network, the tracker can not only obtain semantic information from high-level convolutional features, but also the local features such as texture can be acquired from low-level convolutional features. Therefore, a common practice is to integrate the features of target image and background image learned from different convolutional layers to obtain richer hierarchical information [14]. However, such integration method for convolutional feature is implicit, Because the information of contained feature may be diluted by high-level features, and it cannot fully express the characteristics of low-level features.

## 3. Analysis of Target Tracking Algorithm Based on Multi-Domain Convolutional Neural Network MDNet

Through experimental analysis, it can be found that in the field of savvy transportation, the mind boggling foundation, enlightenment change, impediment and scale change will all influence the following aftereffects of moving focuses in video sequences. The difficulty of tracking targets lies in how to overcome the above-mentioned factors that cause the deterioration of tracking results, on this basis, the similarity problem is solved, and the accuracy and robustness are ensured. In a complex environment, target tracking requires both accuracy and accuracy, but also insure the real-validity. And if occlusion occurs, the tracking result will gradually deteriorate [15]. Therefore, in order to obtain a more robust tracking result, In this paper, improvement of target tracking using multi-domain convolution neural network, The principle work is to consolidate the case division technique with MDNet following calculation. By adding two consideration instruments to the calculation , it extricates better highlights and guarantees that the objective adjusts to the appearance change and significantly further develops the following exhibition. In addition, finally, this method is compared with the existing mainstream methods, which proves its good tracking effect.

### 3.1. Framework of Algorithm

Opromolla et al. proposed a target algorithm with less training data and lower precision is pred in this paper [16]. In 2019, the SiamRPN++ algorithm proposed by Bo Kang et al. combined the deeper neural network with the twin network, which further promoted the development of visual tracking and solved the problem of applying the deep network to target tracking. The MDNet algorithm network consists of a fully connected layer and a domain-specific layer. Offline pretraining shallow layer networks [17]. A new convolution neural network layer based on pretraining, a new two-class layer is used to construct the network, thus improving the tracking efficiency in force.

The methods above still have great drawbacks in solving practical problems. Improved MDNet algorithm improves the robustness of target tracking, and solve the problem of target similarity. In this algorithm, based on the characteristics of convolutional neural network, large data sets are trained off-line before target tracking, so that the initial target tracking algorithm can process predefined objects. On the basis of MDNet tracker, two attribute models (spatial attribute and channel attribute) are added to reduce the influence of attribute difference on attribute learning. On this basis, a method based on multi-domain learning is proposed, which separates domain-related information from domain-related information and combines it with instance segmentation and target tracking.

Firstly, MDNet based on multidomain convolution algorithm is used to improve the tracking performance by adding two models of spatial attention and channel attention. This method is combined with MaskR-CNN's instance segmentation method. Through instance segmentation, the candidate regions of the tracked object can be reduced effectively, and the candidate regions can be obtained. This method supplements the characteristics of vehicle and adjusts the network precisely, thus improving the tracking of target. Because of using the sample segmentation method, a narrower candidate tracking region can be obtained, so a smaller network structure is adopted. This method can not only separate background from foreground object effectively, but also improve tracking accuracy effectively and eliminate the redundancy of the algorithm.

**Figure 1:**
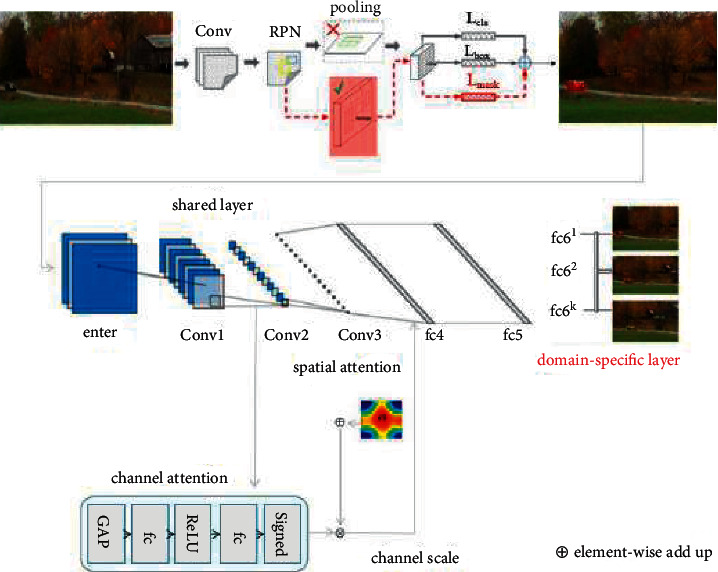
Flowchart of the algorithm in this paper.

Figure 1 shows the basic framework of the tracing algorithm presented in this article. First, the video boundary is segmented using MaskR-CNN, and afterward the up-and-comer areas acquired from the divided pictures are utilized as the contribution of our superior calculation [18]. In this way, the foreground tracking target can be expressed more clearly and tracking range can be reduced. Finally, the network online is fine-tuned during training and testing where the input of the tracker network is an The RGB image size is 107 × 107 and contains the Conv1-Conv3 convolution layer, the fc4-fc5 full connection layer, and two attention components. Although the network size is small, it can still get a better tracking effect.

### 3.2. Instance Segmentation Module

Instance segmentation distinguishes different sample categories according to the representation of pixel feature. Then the targets can be found out in images of different sequence through detection, and then they are classified and regressed to distinguish different instance of the same kind.

#### 3.2.1. Architecture of Mask R-CNN

**Figure 2:**
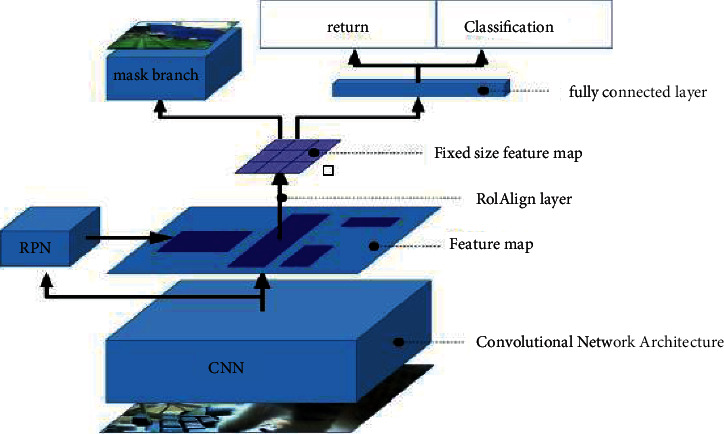
Mask R-CNN network structure.

Based on FasterR-CNN, the overall architecture of MaskR-CNN is extended [19]. As shown in Figure 2, the whole experiment of instance segmentation includes three tasks: classification, regression and segmentation. The framework consists of two parts. The first part is convolution of scanning images to extract different features. The second part is to generate bounding box and mask. When pixel-level tasks are handled in Mask R-CNN, different kinds of targets have different pertinence, which is why it is applied to the tracking algorithm in this paper.

**Figure 3:**
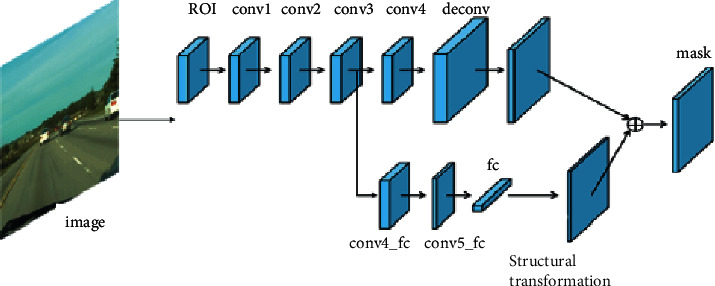
Mask generation flow chart.

As shown in Figure 3, firstly, the data is marked on the video frame; Secondly, the selected RO I is input to the RPN network for secondary classification. Then filter out the unwanted RoI and narrow it down. The RPN network outputs RoI coordinates in the form of [*x*, *y*, *w*, *h*], and then puts them into the RoI library to get a 7 × 7 -size feature map. Finally, the Roi Pooling locale is handled through the Roi Align run. The Roi Align layer will get appropriate fixes, and loss of mean binary cross entropy is adopted in the training of MaskR-CNN algorithm, which can be described as:

(1) $$\begin{array}{c} L_{\text{final}}=L\left(\left\{P_{i}\right\},\left\{t_{i}\right\}+\left(L_{cls}+L_{\text{box}}+L_{\text{mask}}\right)\right), \end{array}$$

where, *L*_*cls*_ and *L*_box_ are used for classification and regression, *L*_mask_ represents the partial loss value of Mask, it includes the size of the output *K* × *m* × *m*, which represents the size of the associated region produced in the image. To maintain area *m* × *m*, it is necessary to ensure that the feature map of the region of interest is aligned with the original picture. Finally, the mean value of the cross entropy of all candidate regions is Lmask.

#### 3.2.2. Network Training

**Figure 4:**
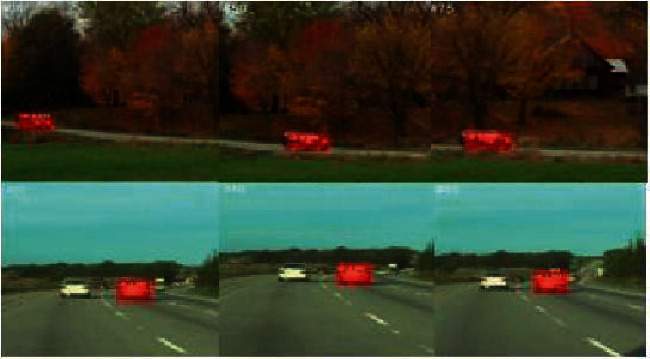
Segmentation result.

In the training stage, each batch of GPU trains two pictures, and positive and negative samples, are classified according to the proportion [20]. Follow-up operation zooms the training pictures according to the set pixel level, samples *n* candidate regions for each picture in the training, and Set the number of positive and negative samples at a ratio of 1 : 3. Do this one time before each practice. Stochastic disturbance to the training data can improve the convergence of the tracking model and improve the model of the test set.  Figure 4 shows the output of MaskR-CNN, which can be seen that even with many complicated challenges, MaskR-CNN still present excellent segmentation.

### 3.3. Attention Mechanism Module

The attention model (AM) is an important part in neural network structure [21], which is widely used in artificial intelligence related processing field [22]. It can learnt from the human visual system for explanation that when people observe a certain scene, the target range is the whole visible scene, but when it is necessary to judge a certain target more deeply, people's attention is focused on the target [23].

The main reasons why attention mechanism is widely used are as follows: (1) Attention model can well solve problems of multitasking, such as semantic translation and image analysis. (2) Improve the feature extraction performance of target tracking algorithm and improving performance of convolution neural network. (3) Overcome the challenge of recurrent neural network well, and have operate better in image classification, target detection and other tasks [24]. Therefore, spatial attention and channel attention are applied to guide the task.

Attention mechanism is to transform the spatial original picture information, and save keywords information through attention mechanism. By finding out the regions that need attention in the image information, the important information of the local image is extracted [25]. TVary with input, preprocessing that suitable for tracking tasks can be completed which can be described as:

(2) $$\begin{array}{c} E\in R^{C\times H\times W}:E_{j}=\alpha \sum_{i=1}^{N}\left(S_{ji}D_{i}\right)+A_{j}, \end{array}$$

where *α* is the scaling coefficient, *S*_*ji*_ is the influence of the feature of the *i*th position on the *j*th position, and *D* is the feature block obtained in convolution.

The channel attention mechanism can be described as an image sequence represented by three channels(R, G, B), where each image channel changes after corresponding convolution. The image matrix of the new channel is transformed that W and H indicate width and height of the image. Moreover, in the module, dot multiplication and Fourier transform are performed on the height and width to decompose the signal of image channel. It can be describe as formula (3):

(3) $$\begin{array}{c} E\in R^{C\times H\times W}:E_{j}=\beta \sum_{i=1}^{c}\left(X_{ji}A_{i}\right)+A_{j}, \end{array}$$

where *β* represents the scale coefficient, which is initialized to 0, *E* represents the feature of a individual channel, and A is the weight between features of channels.

**Figure 5:**
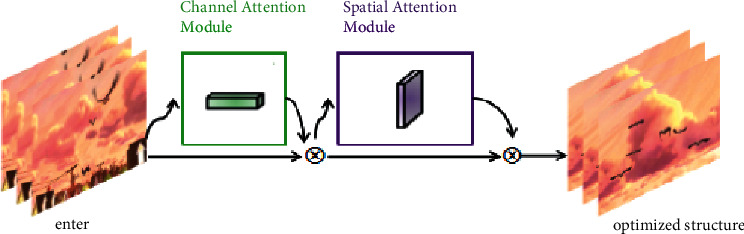
Structure of attention mechanism.

In this paper, the structure of attention mechanism is shown in Figure 5. In computer vision, channel attention mechanism is regarded as a process of extracting various features from semantic association. In practice, the goal is to keep the adaptive deformation and overcome the problem of target similarity, which can greatly improve the tracking efficiency and obtain excellent features by associating channel attention and spatial attention mechanism.

### 3.4. Tracking Module

**Figure 6:**
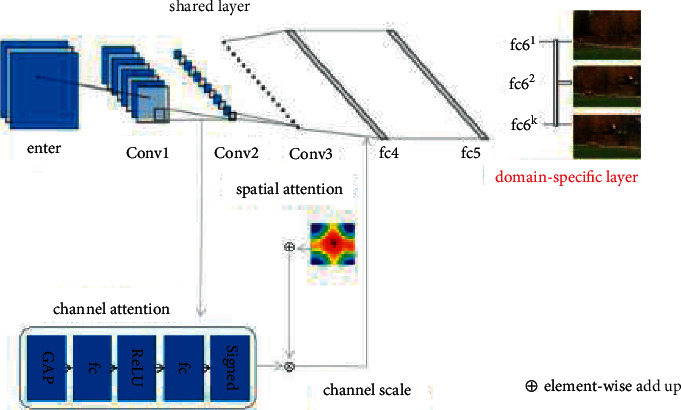
Structure of tracking module.

The principle undertaking of target following is to recognize foundation and closer view focuses in video groupings, which is moderately less complex than general objective arrangement or target acknowledgment [26]. Therefore, the algorithm adopts a simpler network model that is separated from VGG-M [27]. The structure of tracking module is shown in Figure 6. Before tracking the target, The target is segmented by MaskR-CNN algorithm, and the candidate regions of vehicle target are obtained. Usually, as the network develops further, the multidimensional information of the tracked object will be diluted, which will result in serious consequences or even failure. Simultaneously, the element of the competitor locale got by example division is a lot more modest than that of the original image, which constributes to excellent tracking results.

Although attention mechanism module has great influence on tracking results, overlapping attention mechanism module will lead to the performance degradation of the model. A module mechanism of space and channel focus is added to the tracking algorithm, and the external information is used to improve the influence on the robustness of the whole tracking algorithm [28]. Formula (4) is used to code the general learning of all training samples:

(4) $$\begin{array}{c} f_{1}\left(q_{t},K,V\right)=\sum_{s=1}^{m}a\left(q_{t},k_{s}\right)v_{s}, \end{array}$$

There is a query vector t, *K* is the critical matrix, and *V* is the value matrix.

In the process of target tracking, simple network architecture is always adopted. Learning from the excellent target detection method and the idea of Feature Pyramid Networks(FPN), the moving target is tracked with large-scale feature map after convolution [29]. And self-adaption gradient descent method (SGD) is taken to train convolutional neural network. In batch training, the error of the network model is calculated, and then different domains in the iteration are managed. For each repetition, 8 frames are selected sequentially. Of the 8 frames, 4 positive samples (IoU >0.7) and 12 negative values (IoU <0.5) are obtained for each frame. On this basis, 32 positive numbers and 96 negative numbers are studied.

The tracking uses both long-term and short-term updates. When long-term update is adopted, it is updated according to the set specified interval. However, if the long-term update fails, the score of the positive sample of the predicted target will be less than 0.5, and then the short-term update mode will be adopted. How to predict the target location region in subsequent frames is a difficult problem. One approach is to allocate multiple matching templates score before the current frame in the video sequence *f*^+^(*x*^*i*^), *f*^−^(*x*^*i*^) according to the positive and negative samples stored in our network. Finally, in light of the mean adoption strategy, in video sequence *X*^*∗*^, select the sampling region with the highest score as the optimal tracking object state:

(5) $$\begin{array}{c} X^{*}=\text{arg}\underset{x^{i}}{\text{max}}f^{+}\left(x^{i}\right). \end{array}$$

Using Gaussian distribution theory, 256 candidate regions are generated near the position of the predicted object in the previous frame, and are represented by (*x*, *y*, *w*, *h*). Finally, the candidate frames in the original image are clipped and adjusted to 107 × 107, which is used for model input. Afterwards, the highest score can be obtained by forward propagation in the previously candidate regions. Finally, The candidate region with that high score is randomly selected, and the average of candidate region scores is the score of the tracking target framework. Moreover, in the algorithm, 20 positive samples and 100 negative samples according to the borders obtained are generated here, where the positive sample region will be resampled if the number of video frames is larger than 50. Otherwise, if the frame number is larger than 20, the negative sample region will be resampled. In addition, a threshold can be set to compare the calculated score. According to the relationship between the score and threshold, the tracking result is judged.

Use short-term updates in the event of a tracking failure. Select both positive and negative samples of the most recent sequence. First, start 15 rounds of iterative training with the previous iteration. There are 16 positive samples and 512 negative samples randomly selected in each iteration. Then, these 512 negative samples were put into the test model for 4 cycles and the calculated result is the score. Finally, import the training model, calculate the scores of positive samples (16) and difficult negative samples (48) respectively, and track by optimization with calculated parameters.

In order to promote the improved tracking model and avoid the collapse of the target loss function value. The algorithm uses the training curve to find the best learning rate. In the initial training stage, if the setting is too large, the value of target loss function will become larger. RReLU activation function is adopted in the algorithm, which effectively overcomes the gradient disappearance and reduces the over-fitting of the model. In RReLU, aji is uniformly distributed in U(l, U), which will be fixed during the test.

## 4. Experiment of Target Tracking Algorithm Based on Multi-Domain Convolutional Neural Network MDNet

### 4.1. Experimental Platform and Parameters

The configuration of the experimental platform is shown in Table 1.

The input vehicle tracking algorithm is 107 × 107 RGB of images. The network consists of three convolution layers convl-conv3 and two complete connection layers, namely fc4 and fc5. As you can see, this network architecture is smaller than target recognition or target classification networks such as AlexNet, VGG-Net, and so on. Through analyzing the images in each frame, 256 samples in each image are classified, a candidate box with high credibility is selected and tracked. The distribution of the collected samples accords with Gaussian distribution. After unifying their sizes to 107 × 107, they are regarded as the input of the network and then calculated.

The hyperparametric analysis of the model is also carried out. Hyperparameters are variables that are manually set before training, such as a pre-set learning rate. In addition, how to set the learning speed is also very important. In order to meet the requirement of tracing mode, we choose the super parameter. Therefore, the model must be consistent with the actual situation of target tracking. Select a parameter from the maximum to the minimum and give an example of the learning rate. If the average selected value is between [0.0001, 1], 90% of the data comes from [0.0001, 0.1]. Thus, by converting [0.0001, 1] to [−4, 0] (10–4 = 0.0001) and sampling [−4, 0], the value of [0.0001, 0.001] is more likely to reach the maximum learning rate.

### 4.2. Analysis of Results

**Figure 7:**
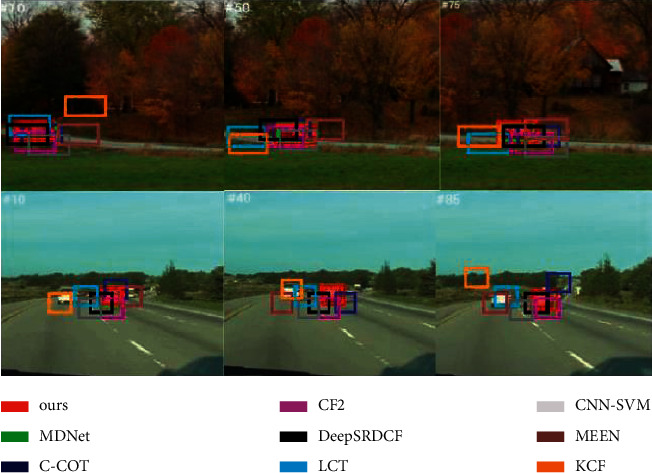
Comparison of experimental results.

On the OTB data set, results in this paper compares with eight advanced target tracking algorithms, including MDNet, C-COT, DeepSRDCF, CF2, CNN-SVM, MEEM, LCT and KCF.  Figure 7 shows the comparison between them:

Through comparative experiments, we can see the improved algorithm can still keep the tracking stable when the target drifts and moves rapidly. At the same time, it can slao capture the target when they are greatly deformed and moving rapidly.

The algorithm proposed in this paper performs well in both measures. Such excellent robust performance is mainly attributed to the fact that the features extracted by attention mechanism is combined with traditional features such as appearance and texture to complement each other, which can converge to a more robust result in training. From Table 2, we can see that this method can realize the intelligent filtering of a certain level of features to a certain extent, effectively suppress noise, effectively solve the problem of vehicle target similarity, and improve the tracking performance of the system.

In the experiment, an independent MDNet target tracking model was first established; Then join the channel attention mechanism module to judge finally, two feature modules are added and segmented with examples, to improve tracking efficiency and solve the problems such as occlusion of vehicle targets. We analyzed the differences between the two models, calculate these indicators by cross-validation method, and evaluate the robustness of the algorithm, in which FIM represents the harmonic average of accuracy and recall rate, and Auc represents its accuracy. The results are shown in Table 3. You can see the accuracy from the comparison, is improved from 90.9% to 90.13% after adding the channel attention mechanism module. In addition The variance decreased from 3.78% to 1.27%, which has better stability. Generally, after adding two attention mechanisms, this method is combined with the image segmentation method, so that it has higher tracking accuracy and better stability.

Furthermore, the robustness and accuracy of video data are explored in different time series, and we cut off the test video in units of length ratio with 1, 2, 3 and 4 , which forms three groups of test data. The results are shown in Table 4 as follows: the shorter the video sequence is, the better the tracking performance is, and the longer the video sequence is, the better the tracking effect. From the above analysis, it can be known that long video sequences are of great significant to our training tracker.

## 5. Conclusion

Target tracking is a very important application in computer vision, which has a very wide prospect. Target tracking can be widely promoted in smart traffic supervision, public safety monitoring, unmanned driving and other fields. Because of the outstanding performance of deep learning related technologies in target detection and classification, many researchers have introduced deep learning technology into the field of target tracking. Therefore, in this paper, the instance segmentation algorithm is used to segment the target to be tracked, which is used as the input of the target tracker. [30–32].

## Figures and Tables

**Table 1:** Configuration of experimental platform used for algorithm simulation.

Configuration content	Model
Processor model	2.30 GHz Intel (R) Xeon (R) gol *d* 5118
Storage configuration	RAM 16G, 512 G SSD
Graphics card configuration	NVIDIA GTX 1070
Operating system	Windows
Software platform	Pycharm
CUDA	Cuda8.0.61 for ubuntu 16.04
Deep learning platform	Tensorflow1.0

**Table 2:** Comparison of experimental data.

s	Accuracy	Success rate
Algorithm	0.918	0.678
MDNet	0.909	0.678
C-COT	0.898	0.671
DeepSRDCF	0.854	0.635
CF2	0.837	0.562
CNN-SVM	0.814	0.562
MEEM	0.781	0.554
LCT	0.762	0.530
KCF	0.692	0.475

**Table 3:** Comparison of different models.

Method	Accuracy (%)	Variance (%)	FIM (%)	GM (%)	Auc (%)
MDNet	90.90	3.78	82.10	85.04	67.80
Add channel attention module	90.13	1.27	83.50	87.09	67.80
Algorithm	91.80	0.57	86.79	89.37	67.80

**Table 4:** Comparison of the results of different video sequences.

Video sequence	Accuracy (%)	Variance (%)	FIM (%)	GM (%)	Auc (%)
1	78.35	4.12	77.19	75.21	46.18
2	87.51	2.70	78.35	80.68	59.88
3	89.60	0.66	83.99	87.28	62.98
4	91.80	0.57	86.79	89.37	67.80

## Data Availability

The dataset can be accessed upon request.
